# Supplementary material for: 17βH-Neriifolin Improves Cardiac Remodeling Through Modulation of Calcium Handling Proteins in the Heart Failure Rat Model
Source: Biomedicines. 2025 Aug 29;13(9):2115. doi: 10.3390/biomedicines13092115 (PMC12467975; doi:10.3390/biomedicines13092115)
Supplement: Supplementary file 1 [file biomedicines-13-02115-s001.zip › biomedicines-3768173-supplementary.pdf]

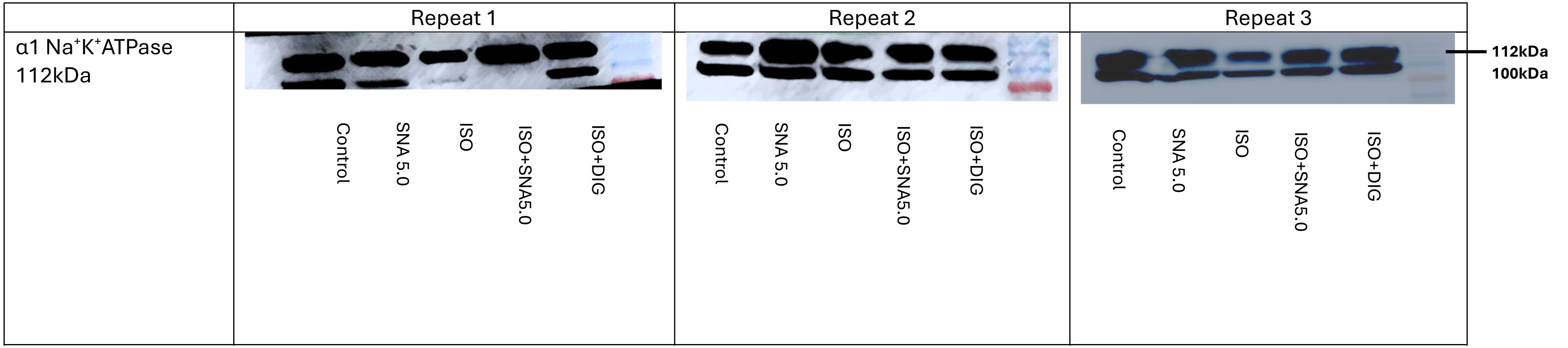
Repeat 1
Repeat 2
Repeat 3
α1 Na+K+ATPase 112kDa
Control
SNA 5.0
ISO
ISO+SNA5.0
ISO+DIG
112kDa
100kDa
(a)

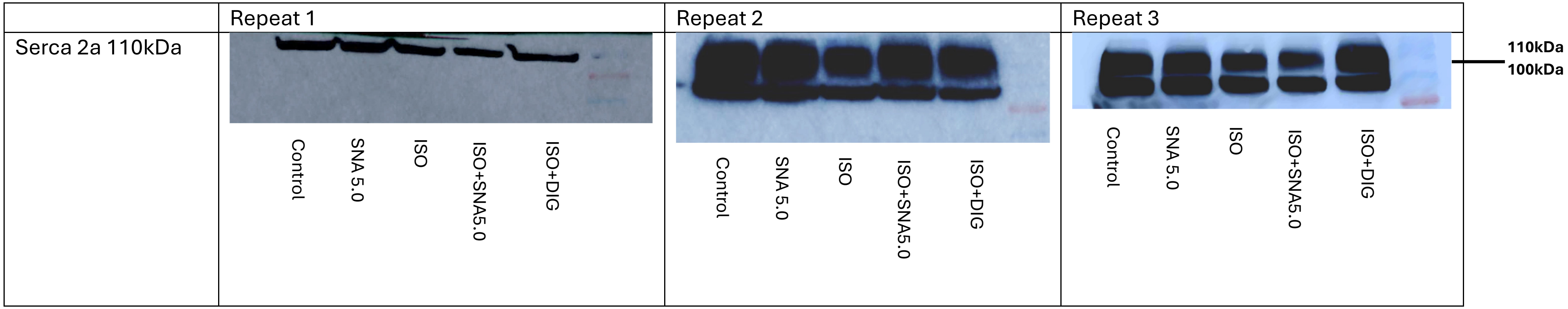
Repeat 1
Repeat 2
Repeat 3
Serca 2a 110kDa
Control
SNA 5.0
ISO
ISO+SNA5.0
ISO+DIG
110kDa
100kDa
(b)

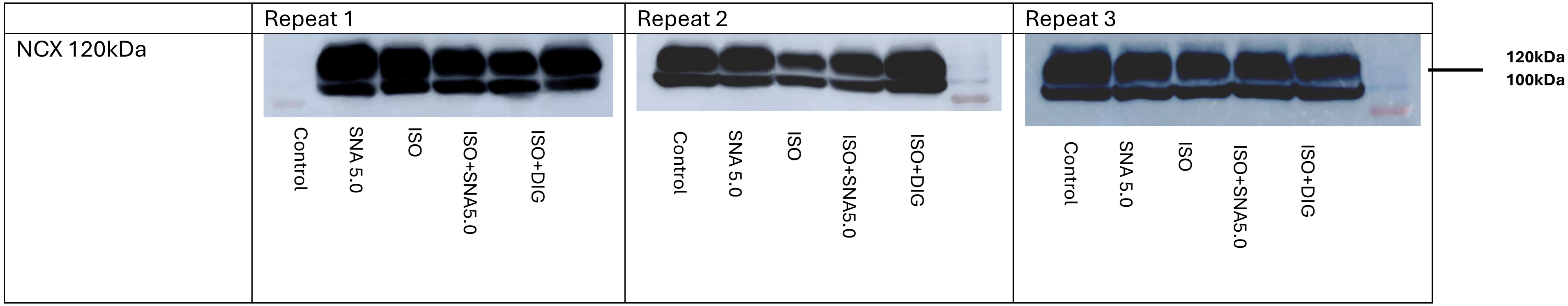

(c)

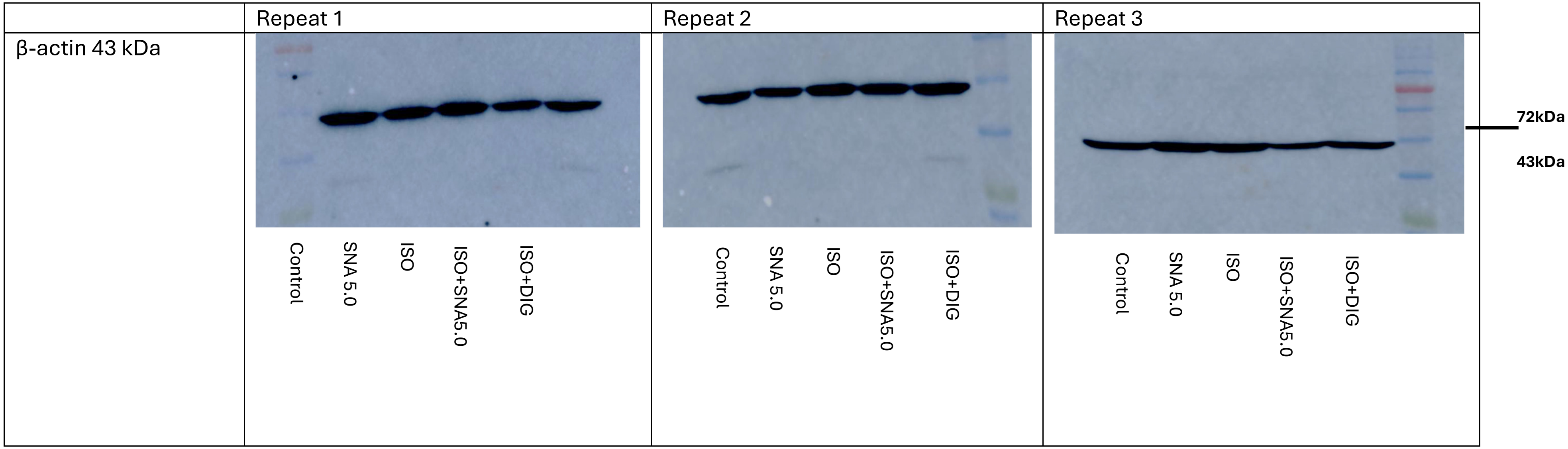

(d)

Figure S1. a shows the whole blot, which shows one band at α1 Na+K+ATPase (112kDa). The screen was brightened, and the marker of the protein ladder was dimmed. b shows the whole blot, which shows one band at SERCA 2a (110kDa). The screen was brightened, and the marker

of the protein ladder was dimmed. c shows the whole blot showing one band at NCX (120kDa). The screen was brightened, and the marker of the protein ladder was dimmed. d shows the whole blot that shows one band at β-actin (120kDa), the screen was brightened and the marker of protein ladder was dimmed.
